# The co-expression of MMP-9 and Tenascin-C is significantly associated with the progression and prognosis of pancreatic cancer

**DOI:** 10.1186/s13000-015-0445-3

**Published:** 2015-12-10

**Authors:** Yingqiang Xu, Zhonghu Li, Peng Jiang, Guo Wu, Kai Chen, Xi Zhang, Xiaowu Li

**Affiliations:** Department of Hepatobiliary Surgery Institute, Southwest Hospital, Third Military Medical University, Chongqing, China; No.30 Gaotanyan Street, Shapingba District, Chongqing, 400038 China

**Keywords:** MMP-9, TN-C, ECM, Prognosis, Pancreatic cancer

## Abstract

**Background:**

Matrix metalloproteinase-9 (MMP-9) and Tenascin-C (TN-C) have been shown to be involved in the metastasis of many tumors. The purpose of this study was to determine the relationship between the co-expression of these two molecules and the clinical prognosis of pancreatic cancer.

**Methods:**

We investigated the expression of TN-C and MMP-9 in 103 pancreatic cancer tissues by immunohistochemistry and used statistical analyses to investigate the correlations of individual expression or co-expression of these two molecules with clinicopathological parameters and survival of pancreatic cancer.

**Results:**

The expression of MMP-9 and TN-C were increased in pancreatic cancer. The co-expression of MMP-9 and TN-C was also detected. The expression of MMP-9 and TN-C were correlated with vascular invasion, lymph node invasion, liver metastases and TNM stage. The co-expression of MMP-9 and TN-C was significantly related to the pancreatic cancer metastases. The individual overexpression of MMP-9 or TN-C significantly decreased the overall survival rates. The co-expression of MMP-9 and TN-C had the lowest overall survival rates. The co-expression of MMP-9 and TN-C was an independent predictor of survival for pancreatic cancer patients.

**Conclusions:**

Co-expression of MMP-9 and TN-C was associated with poorer prognosis and was found to be an independent predictor of survival.

## Background

Pancreatic cancer is one of the most aggressive and metastatic malignant tumors [1]. Previous studies have shown that various extracellular matrix (ECM) proteins are involved in the cancer progression and prognosis. However, the role of the ECM proteins on the prognosis of patients with pancreatic cancer remains unclear.

The ECM is well known for playing a key role in inflammation, lesions, tissue repair, tumor invasion and metastasis formation [2, 3]. Numerous proteins that constitute the ECM show a close association with tumor tissue, such as Tenascin-C (TN-C) and Matrix metalloproteinases (MMPs) [4, 5]. TN-C is a complex multifunctional protein, which can influence cellular behavior directly via cell surface receptors or indirectly by binding to other matrix proteins [6, 7]. This induces angiogenesis and promotes cell migration [8]. The fibrillar TN-C (fTN-C) is primarily expressed in tumor extracellular matrix, and fTN-C matrix formation requires the participation of MMPs and may play a role in promoting cancer metastasis [4, 9]. TN-C overexpression can impact the progression and prognosis of various malignant tumors, including glioma [10], gastric cancer [11], breast carcinoma [12], and Merkel cell carcinoma [13]. However, the role of TN-C on the prognosis of patients with pancreatic cancer remains unclear. MMPs are a group of zinc-binding endopeptidases and can degrade multiple components of the ECM and regulate ECM remodeling [5]. MMPs are associated with cancer growth and are considered as the prime candidates during tumor invasion, metastasis and angiogenesis [14, 15]. Degradation of the ECM is an essential step in tumor invasion and metastasis, and the MMP-9 was able to degrade the ECM and promote tumor cell metastasis. In previous studies, MMPs have been shown to be involved in TN-C formation, but the relationship of MMP-9 and TN-C in pancreatic cancer is still not clear.

Although TN-C and MMP-9 have been thought to be related to invasion and metastasis of pancreatic cancer [9, 16], it is unclear whether there is a co-expression of MMP-9 and TN-C in pancreatic cancer. In this study, we investigated the levels of TN-C and MMP-9 in pancreatic cancer tissues by immunohistochemistry and analyzed the correlations of the individual expression of MMP-9 and TN-C with clinicopathological parameters and survival of pancreatic cancer patients using statistical analysis methods. Then, we investigated the relationship between the co-expression of these two molecules and the clinical prognosis of pancreatic cancer.

## Methods

### Patients

A total of 103 Chinese patients (67 males and 36 females) with a median age of 56 years (range 36-86 years) underwent surgery at the Department of Hepatobiliary Surgery Institute, Southwest Hospital, Third Military Medical University, China, for pancreatic cancer from January 2007 to June 2010. All patients underwent curative resection by pancreaticoduodenectomy and pylorus-preserving pancreaticoduodenectomy with lymph node dissection. Patients who had received neoadjuvant or adjuvant radio or chemotherapy were excluded in this study! All of the tumors were invasive ductal adenocarcinomas histologically, including 9 (8.7 %) well-differentiated, 67 (65 %) moderately differentiated and 27 (26.2 %) poorly differentiated cases, 15 (14.5 %) tumors were found in head of pancreas and 88 (85.5 %) in body. Vascular invasion and lymph node metastasis were observed in 36 (34.9 %) tumors and 41 (39.8 %), respectively. The number of patients with pT1, pT2, pT3, and pT4 tumors was 17 (16.5 %), 31 (30.1 %), 51 (49.5 %), and 4 (3.9 %), respectively. All patients were assessed by ultrasonography, radiography and computed tomography every 3 months after discharge. New lesions detected by imaging were considered indicative of relapse. The median follow-up period was 13 months (range 3-49 months), the median survival was 11 months. During this period, 13 patients experienced recurrence of liver disease, 1 patient experienced recurrence of lung disease, 91 patients were dead. In addition, 6 normal pancreatic samples were collected from the donors for liver transplantation.

This study was approved by the Ethics Committee of the Southwest Hospital, and all patients provided written informed consent.

### Immunohistochemistry

A total of 103 pancreatic cancer specimens were fixed in formalin, embedded in paraffin and cut into 3- micrometer serial sections. The deparaffinized sections were trypsinized (0.05 % trypsin with 0.05 % Triton X-100 in TBS) for 20 min. Slides were heated at 120 °C in an autoclave in 10 mM sodium citrate (pH 6.0) for 130 s and cooled to room temperature. After blocked with 10 % goat serum in Superblock, each section was incubated separately with anti-TN-C (Sigma; HPA004823) at 20 μg/ml and anti-MMP-9 (Proteintech; 10375-2-AP) at 8 μg/ml at 4 °C for 18-24 h. The sections were incubated with anti-mouse/rabbit immunoglobulins (Dako EnVisionTM System; K5007) for 60 min at 37 °C. After washing four to five times (15 min each) with Triton-TBS, the slides were processed in the Ventana-automated stainer according to the manufacturer's instructions. The immunoperoxidase-3, 3-diaminobenzidine-stained slides were subsequently counterstained with hematoxylin and mounted with a coverslip.

**Fig. 1:**
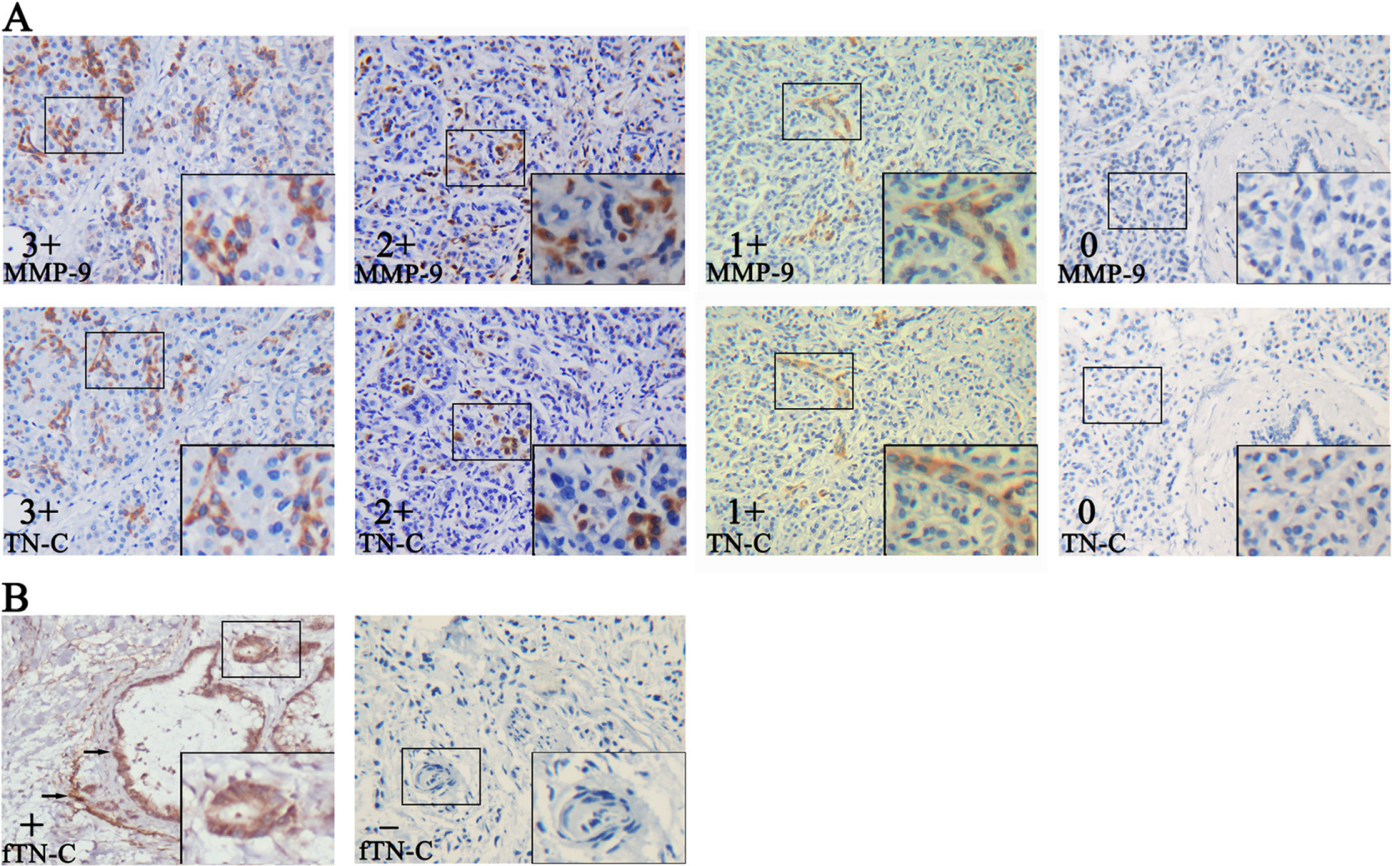
Immunohistochemical staining of MMP-9 and TN-C in pancreatic cancer. **a** Upper panels are MMP-9 immunostaining from 3 to 0, respectively; lower panels are TN-C immunostaining from 3 to 0, respectively. **b** fTN-C stain was positive (left) and negative (right), respectively; the arrow is a tube-shaped fTN-C. Original magnification, 200 ×, 400 × magnification was showed in the bottom right box

The immunostained sections were evaluated by two independent investigators without knowledge of the patients’ identity and clinical status. The investigators that scored the sections are trained pathologist. Ten random fields were selected, and expression was evaluated in 1,000 tumor or stromal cells (100 cells per field) with an image analyzer (MetaMorph Imaging System version 6.0), then the slide were scored as 0, 1, 2, 3 and 4 if the percentage of positive cells were less than 5 %, 6-25 %, 26-50 %, 51-75 % and 76-100 %, respectively. The staining intensity of MMP-9 and TN-C was scored as 0, 1, 2 and 3 according to the intensity of positive staining color. In the end, the final score was evaluated by the multiplications of the scores of positive cells and intensity of positive staining: 1+ (multiplication 1-4), 2+ (multiplication 5-8) and 3+ (multiplication 9-12) (Fig. 1a; Table 1). The staining of fTN-C was further determined based on the fibril shape of TN-C. The staining intensity of fTN-C was positive and negative respectively (Fig. 1b). For statistical analysis and to reduce intraobserver variability, the immunohistochemical scores were further grouped into the following two categories: negative (grade 0 or 1+) or positive (grade 2+ or 3+).

**Table 1:** Expression of MMP-9, TN-C, fTN-C and their co-expression in the pancreatic cancer samples (n=103)

	Expression level					
	0	1	2	3	-	+
MMP-9	12	30	19	42	42	61
TN-C	26	21	28	28	47	56
fTN-C					75	28
MMP-9 + TN-C					60	43

“*0-3*”was refer to the staining score, “*-*”: negative, “*+*”: positive

### Statistical analyses

All statistical analyses were performed using the IBM SPSS Statistics 19.0 software. Group differences were statistically analyzed using the χ^2^ test. Correlation of TN-C with MMP-9 was calculated by Pearson χ^2^ test. The Kaplan-Meier method was used to analyze survival and the log-rank test was used to estimate differences in survival. Prognostic factors were examined using univariate and multivariate analyses, the multivariate analysis by a Cox proportional hazards regression model with a Forward LR method. All reported P values were two-sided, and *P* < 0.05 was considered statistically significant. All statistical analyses were completed under the guidance of experienced experts in the Statistics Department.

## Results

### The co-expression of MMP-9 and TN-C in pancreatic cancer

MMP-9 and TN-C have been associated with tumor invasion and metastasis. To determine the relationship between pancreatic cancer and the expression of MMP-9 and TN-C, we analyzed the expression levels of MMP-9 and TN-C in 103 pancreatic cancers and 6 normal pancreatic samples by immunohistochemistry. We found that the expression of MMP-9 (stain ratio: 88 %) and TN-C (stain ratio: 74 %) was increased in most of pancreatic cancers compared with normal pancreatic samples (stain ratio: 0). MMP-9 and TN-C is primarily overexpressed in the stroma but exists in part in the cytoplasm of tumor cells. We also detected the MMP-9 and TN-C can be expressed in the same tumor region through serial section, and identified as co-expression (Fig. 1). Moreover, the pancreatic cancer samples that experienced recurrence of liver metastases had a stronger expression of MMP-9 and TN-C. Of the 103 pancreatic cancers, the expression levels of MMP-9 were high in 61 samples and low in 42 samples, and the expression of TN-C was high in 56 samples and low in 47 samples (Table 1). In addition, as shown in Table 2, the expression levels of MMP-9 were significantly correlated with TN-C in 103 pancreatic cancer specimens (*P* < 0.001). Interestingly, a tube-shaped fTN-C was found in 28 samples, primarily focused on the vascular invasion, lymph node and liver metastases samples (Fig. 1, Table 1). Furthermore, the co-expression of MMP-9 and TN-C was positive in 43 samples, and 12 samples had hepatic metastasis (Table 1). These results suggest that the expression of MMP-9 and TN-C is increased in pancreatic cancer and that the co-expression of these two molecules may be associated with the distant metastasis of pancreatic cancer.

**Table 2:** MMP-9 and TN-C protein levels correlated in 103 pancreatic cancer tissues

	MMP-9		
	Negative	Positive	P-value
TN-C			<0.001
Negative	29	18	
Positive	13	43	

### Co-expression of MMP-9 and TN-C was correlated with lymph node metastasis, vascular invasion and hepatic metastasis

To investigate whether MMP-9 and TN-C are involved in the metastasis of pancreatic cancer, we further analyzed the relationship between the expression levels of MMP-9 and TN-C and the clinical characteristics of the pancreatic cancer patients. As shown in Table 3, the overexpression of individual MMP-9 or TN-C was correlated with lymph node metastasis (*P* = 0.019, χ^2^ = 5.49 and *P* = 0.021, χ^2^ = 5.23), vascular invasion (*P* = 0.049, χ^2^ = 3.87 and *P* = 0.024, χ^2^ = 5.07), hepatic metastasis (*P* = 0.009, χ^2^ = 6.47 and *P* = 0.003, χ^2^ = 8.63) and TNM stage (*P* = 0.019, χ^2^ = 13.56 and *P* = 0.012, χ^2^ = 14.73). The co-expression of MMP-9 and TN-C was correlated with lymph node metastasis (*P* < 0.001, χ^2^ = 13.15), vascular invasion (*P* = 0.003, χ^2^ = 8.53), hepatic metastasis (*P* < 0.001, χ^2^ = 15.64) and TNM stage (*P* < 0.001, χ^2^ = 25.06). The formation of fTN-C was involved in lymph node metastasis (*P* = 0.008, χ^2^ = 7.02), vascular invasion (*P* = 0.015, χ^2^ = 5.86), hepatic metastasis (*P* = 0.003, χ^2^ = 8.87) and TNM stage (*P* = 0.005, χ^2^ = 16.74). These results suggested that the co-expression of MMP-9 and TN-C are correlated with lymph node metastasis, vascular invasion and hepatic metastasis in pancreatic cancer.

**Table 3:** Clinicopathologic correlation of MMP-9 and TN-C status in 103 pancreatic cancer

	MMP-9			TN-C			fTN-C			MMP-9 + TN-C		
	-	+	*P*	-	+	*P*	-	+	*P*	-	+	*P*
Gender			0.480			0.066			0.573			0.096
Male	29	38		35	32		50	17		43	24	
Female	13	23		12	24		25	11		17	19	
Age, years			0.416			0.588			0.582			0.692
<65	32	42		35	39		55	19		44	30	
≥65	10	19		12	17		20	9		16	13	
Tumor location			0.229			0.636			0.227			0.676
Head	38	50		41	47		66	22		52	36	
Body/tail	4	11		6	9		9	6		8	7	
Tumor size, cm			**0.032**			0.305			0.738			0.06
≤2	18	14		17	15		24	8		23	9	
>2	24	47		30	41		51	20		37	34	
Lymphatic invasion			**0.019**			**0.021**			**0.008**			**<0.001**
Negative	31	31		34	28		51	11		45	17	
Positive	11	30		13	28		24	17		15	26	
Vascular invasion			**0.049**			**0.024**			**0.015**			**0.003**
Negative	32	35		36	31		54	13		46	21	
Positive	10	26		11	25		21	15		14	22	
Hepatic metastases			**0.009**			**0.003**			**0.003**			**<0.001**
Negative	41	49		46	44		70	20		59	31	
Positive	1	12		1	12		5	8		1	12	
Differentiation			0.077			0.146			0.429			**0.034**
Poor	7	20		8	19		18	9		10	17	
Moderate	29	38		34	33		49	18		44	23	
Well	6	3		5	4		8	1		6	3	
T-factor (UICC)			**0.021**			0.137			**0.035**			**0.043**
T1	11	6		12	5		15	2		14	3	
T2	16	15		13	18		25	6		20	11	
T3	14	37		21	30		34	17		25	26	
T4	1	3		1	3		1	3		1	3	
Stage (UICC)			**0.019**			**0.012**			**0.005**			**<0.001**
1A	7	2		8	1		9	0		9	0	
1B	12	8		9	11		19	1		15	5	
2A	10	16		15	11		19	7		19	7	
2B	10	22		12	20		22	12		14	18	
3	1	1		1	1		2	0		1	1	
4	2	12		2	12		6	8		2	12	

The bold values indicate P-values less than 0.05, *UICC* Union for International Cancer Control

### Co-expression of MMP-9 and TN-C indicates a poorer prognosis in pancreatic cancer patients

**Fig. 2:**
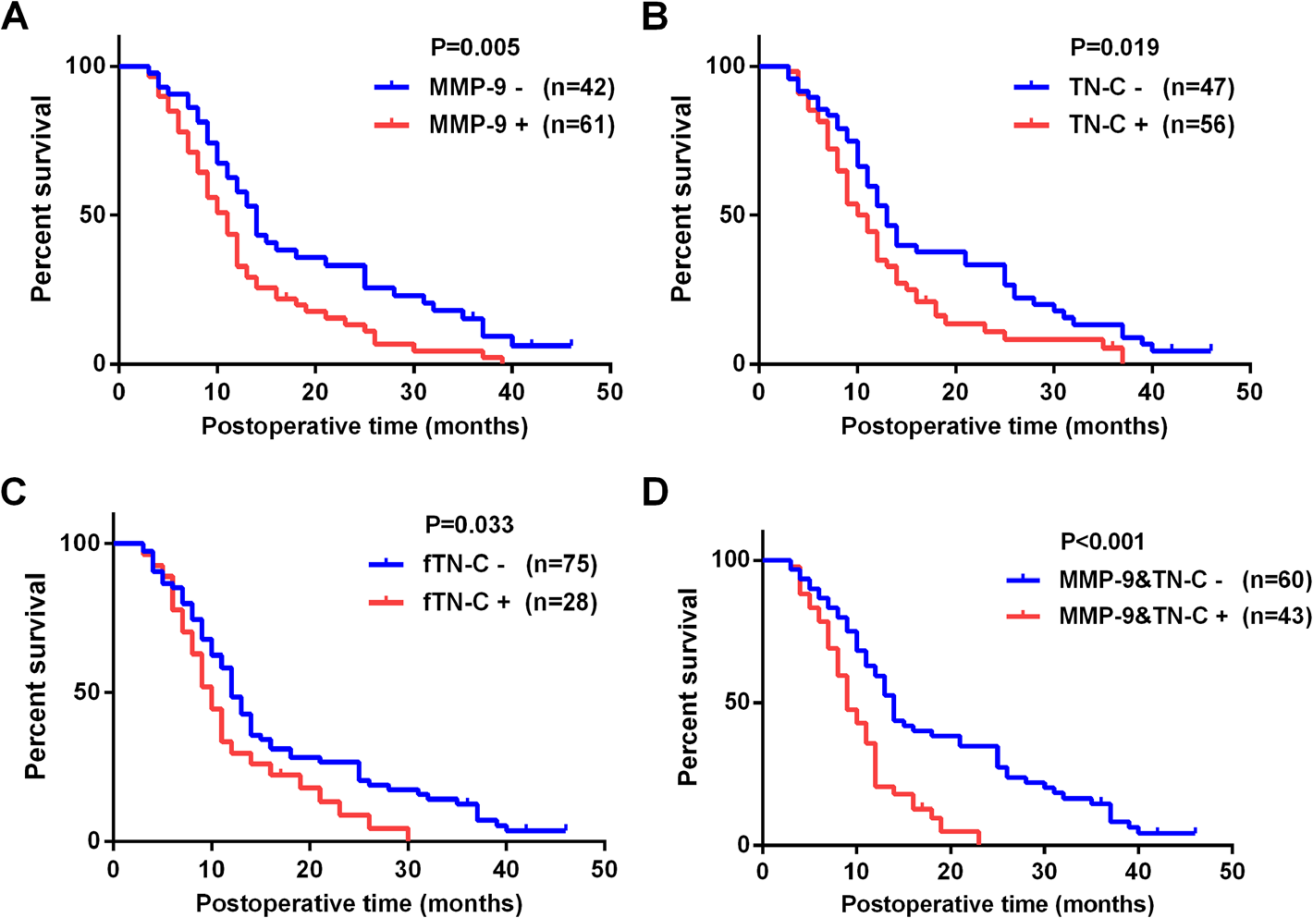
Kaplan-Meier survival curves for overall survival of patients with pancreatic cancer according to the expression status of (**a**) MMP-9, (**b**) TN-C, (**c**) fTN-C, (**d**) Co-expression of MMP-9 and TN-C

To determine the effects of MMP-9 and TN-C on the survival rates of pancreatic cancer patients, we used Log-rank test analyses. Kaplan-Meier survival curves showed that the individual overexpression of MMP-9, TN-C or fTN-C significantly decreased overall survival (*P* = 0.005, *P* = 0.019, *P* = 0.033, respectively) (Fig. 2a-c). The median survival of individual overexpression of MMP-9, TN-C or fTN-C were 11 months, 11 months, 10 months separately, the median survival of co-expression of MMP-9 and TN-C was 9 months. Furthermore, compared to individual overexpression of MMP-9, TN-C or fTN-C, the co-expression of MMP-9 and TN-C had the lowest overall survival and 1-year survial rate (*P* < 0.001) (Fig. 2d, Table 4).

**Table 4:** Median survivals and 1 year survival rates of MMP-9, TN-C, fTN-C and their co-expressions

Variables	Median survial time (month)	1-year survial rate (%)	
		Negative	Positive
MMP-9	11	59.3%	32.2%
TN-C	11	54.4%	34.1%
fTN-C	10	58.4%	29.7%
MMP-9+TN-C	9	59.4%	20.4%

### Co-expression of MMP-9 and TN-C is an independent predictor of survival in pancreatic cancer

To determine the independent risk factors of survival, we used univariate and multivariate analyses. We found that the individual expression or co-expression of MMP-9 and TN-C were risk factors affecting survival of patients by the univariate analysis. In contrast, multivariate analysis showed that only the co-expression of MMP-9 and TN-C was an independent predictor for survival of patients (*P* = 0.042) (Table 5).

**Table 5:** Univariate and multivariate Cox regression of prognostic factors for overall survival in pancreatic cancer

	Univariate P	Multivariate P	Hazard ratio	95% Confidence interval
pL	0.011	0.922		
Negative/Positive				
pV	0.016	0.742		
Negative/Positive				
Hepatic metastases	0.043	0.116		
Negative/Positive				
pT	<0.001	0.123		
T1,2/ pT3,4				
Differentiation	0.011	0.201		
Poor/ Moderate/ Well				
pStage	<0.001	**0.010**	1.642	1.124-2.400
I / II / III,IV				
MMP-9 expression	0.005	0.938		
Negative/Positive				
TN-C expression	0.019	0.766		
Negative/Positive				
fTN-C expression	0.033	0.978		
Negative/Positive				
MMP-9, TN-C expression	<0.001	**0.042**	1.727	1.019-2.927
Negative/Positive				

The bold values indicate P-values less than 0.05. *pL* lymphatic invasion, *pV* pathological vessel status, *pT* T-factor, *pStage* TNM stage (UICC)

## Discussion

The extracellular matrix (ECM) plays an important role in tumor growth, invasion and metastasis [17]. Our previous study demonstrated that fTN-C matrix formation required participation of MMPs and plays a role in promoting cancer metastasis [9]. In this study, we only focus on the relation of MMP-9, total TN-C and fTN-C in pancreatic cancer invasion, we found that the expression of MMP-9 and TN-C was increased in all pancreatic cancer tissues compared with normal pancreatic tissue. In fact, TN-C was primarily overexpressed in the stroma but also existed in the cytoplasm of tumor cells. This finding was consistent with previous research results [18, 19]. Present studies have shown that MMPs not only induce TN-C expression but also promote the formation of fTN-C matrices. In 103 pancreatic cancer specimens, 43 samples exhibited the co-expression and co-location of MMP-9 and TN-C. In addition, the MMP-9 expression was significantly correlated with TN-C expression, this co-expression and co-location of MMP-9 with TN-C in pancreatic cancer may imply that MMP-9 helps TN-C incorporate into the stroma to form tube-shaped structures named “tubular matrix channels” or “vasculogenic mimicry”, which guide tumor cells to migrate to vascular invasion [20]. It should be noted that cancer-fibroblast cell interaction leads to a “fibrillar” organization of extracellularely deposited Tn-C, However, this is not a real fibrillogenesis but is due to a molecular interaction of tenascin-C with other molecules including fibrillary, therefore we interpret the “fibril like” stromal depositions as “tubular matrix channels” or “vasculogenic mimicry [21–23]. Stromal deposition of TN-C seems to be associated with an “activated” tumor stroma / presence of cancer-associated fibroblasts (CAFs) as indicated by alpha smooth muscle actin (ASMA) positivity [24–26]. Interestingly, in this study, the tube-shaped structures of fTN-C that were found in 28 pancreatic cancer samples were primarily focused on pancreatic cancer with vascular invasion, lymph node and liver metastases. Therefore, the co-expression of MMP-9 and TN-C may promote tumor metastasis and thus affect the progression of pancreatic cancer.

Pancreatic cancer is characterized by aggressive behavior, poor prognosis, and low survival rate [1, 27]. However, whether the expression of TN-C and MMP-9 is involved in the clinical prognosis of pancreatic cancer patients is unclear. Present studies have shown that TN-C is a predictor of survival in colorectal [28], breast [18, 29] and cervical cancers [30], but not in pancreatic cancer [31]. In contrast, the MMP-9 in the prognosis of pancreatic cancer patients is still controversial [32–34]. Especially, Lekstan A found that the formation of lymph node metastases was characterized by the higher concentrations of MMP9 in pancreatic cancer [16], Gardian K also found a high expression of MMP9 at more advanced tumors in a meaningful research about microenvironment elements [35], nevertheless, both of them had an absence of survival analysis. In this study, we found that overexpression of MMP-9, TN-C and fTN-C was correlated with lymph node metastasis, vascular invasion, hepatic metastasis and TNM stage, but not differentiation, this is little inconsistent with previous study by Juuti et al. [31], which shows that TN-C is correlated with differentiation. This inconsistent may be original from that the fTN-C was not distinguished in their study. In addition, the co-expression of MMP-9 and TN-C was significantly associated with lymph node metastasis, vascular invasion and hepatic metastasis. Univariate analysis showed that MMP-9, TN-C, and fTN-C expression levels, co-expression of MMP-9 and TN-C were risk factors and affected patient survival. Multivariate analysis showed that the co-expression of MMP-9 and TN-C was an independent predictor for the survival of pancreatic cancer patients. Kaplan-Meier survival curves showed that the individual overexpression of MMP-9, TN-C or fTN-C significantly decreased the overall survival and that co-expression of MMP-9 and TN-C had the lowest overall survival. Therefore, the co-expression of these two molecules may indicate a poorer prognosis in pancreatic cancer patients.

Our study provided the real close clinical evidences between MMP-9 and TN-C in pancreatic carcinoma, we further showed fTN-C had tube-like structures in 28 pancreatic cancer patients, interestingly, most of them experienced vascular invasion, which suggests its possible functions in angiogenesis or metastasis. The underlying mechanisms and the cellular effects of the co-expressions and fTN-C were worth studying, which would be our next work. However, this study had several limitations. First, this data was from a single-center. Second, this study was retrospective and only patients who underwent curative resection were included. Third, the co-expression study via immunohistochemistry (semi-quantitative) may not precise enough. These questions would be solved in our next study.

In conclusion, we demonstrated that MMP-9 and TN-C were overexpressed in pancreatic cancer and were associated with tumor progression and prognosis. The co-expression of these two molecules was associated with poorer prognosis and was found to be an independent predictor of survival. Combining with previous research, it may provide a novel biomarker and potential therapeutic targets in human pancreatic cancer.

## Conclusions

In this study, via using immunohistochemistry and clinical survival analysis we investigated the expression patterns of TN-C and MMP-9 and their correlations of individual expression or co-expression with clinicopathological parameters and survival of pancreatic cancer.in 103 pancreatic cancer tissues, we found that the elevated expressions of TN-C and MMP-9 were related to the pancreatic cancer metastases and the low survival rates, The co-expression of MMP-9 and TN-C had the lowest overall survival rates, The co-expression of MMP-9 and TN-C was an independent predictor of survival for pancreatic cancer patients.
